# The complete mitochondrial genome of *Rana johnsi* (Smith, 2009) (Anura: Ranidae) and its phylogeny

**DOI:** 10.1080/23802359.2021.2002213

**Published:** 2021-11-29

**Authors:** Qing-Ping Chen, Lian Wu, Shu-Sheng Zhang, Lei-Lei Liu, Wan-Ting Jin, Jia-Yong Zhang, Yong-Pu Zhang, Dan-Na Yu

**Affiliations:** a College of Chemistry and Life Science, Zhejiang Normal University, Jinhua, PR China; b College of Life and Environmental Science, Wenzhou University, Wenzhou, PR China; c The Management Center of Wuyanling, National Natural Reserve in Zhejiang, Wenzhou, PR China; d Key Lab of Wildlife Biotechnology, Conservation and Utilization of Zhejiang Province, Zhejiang Normal University, Jinhua, PR China

**Keywords:** *Rana johnsi*, mitogenome, phylogeny

## Abstract

*Rana johnsi* (Smith 2009) firstly considered as the member of genus *Pseudorana*, has been moved into the genus *Rana*. In this study, we sequenced the complete mitochondrial (mt) genome of *R. johnsi* using the Sanger method. The circular mt genome was 17,873 bp in length and contains 13 protein-coding genes (PCGs), 22 transfer *RNA* (*tRNA*) genes, two ribosome *RNA* genes, and one control region. The overall nucleotide composition in majority-strand was 28% A, 29% T, 29% C, and 14% G. We discussed the phylogenetic relationship of *R. johnsi* in genus *Rana* using ML and BI analyses based on 13 PCGs. Excluding the clade of subgenus *Lithobates*, *Rana draytonii* was the basal clade to all other *Rana* species, which included *R. johnsi* as the basal clade. The monophyly of genus *Rana* was supported, whereas *Pseudorana* was failed to support.

The phylogenetic relationships of *R*ana were involved in a heated discussion using mitochondrial (mt) genomes (Dubois 1992; Frost et al. 2006; Che et al. 2007). *Rana johnsi* (Smith 2009) (Anura: Ranidae), called as John’s groove-toed frog (Fei et al. 1999), was distributed in China, Vietnam, Laos, Thailand, and Cambodia (Frost 2021). Whereas *R. johnsi* was belonging to genus *Pseudorana* according to the taxonomy of Fei et al. (2010). But now it is considered as genus *Rana* species. The mitogenome of *R. johnsi* has not been reported to date. So, in this study, we sequenced and analyzed the mt genome of *R. johnsi* to discuss its phylogenetic relationship.

The frog sample (No. GXJX20180723-1) collected from Jinxiu, Guangxi Province, China (24.14°N, 110.18°E) was identified by JY Zhang and stored at −40 °C in the Animal Specimen Museum, College of Life Sciences and Chemistry, Zhejiang Normal University, China. Total genomic DNA (No. YNGZW0723) was extracted from leg muscle using an Ezup Column Animal Genomic DNA Purification Kit (Sangon Biotech Company, Shanghai, China) and stored in the Zhang’s lab (http://sky.zjnu.edu.cn/2019/0319/c4853a284409/page.htm, DN Yu, email: ydn@zjnu.cn). The mt genome was amplified by polymerase chain reaction (PCR) with universal primers according to Zhang et al. (2013). The specific primers were designed based on the amplified fragments by Primer Premier version 5.0 (Primer Biosoft International, Palo Alto, CA) to amplify the remaining gaps between sequences. The obtained whole mt genome was deposited in the NCBI with accession number MZ571365.

The complete mt genome of *R. johnsi* in majority-strand was 17,873 bp in length with negative AT-skew and GC-skew, which were −0.006 and −0.349, respectively. It encoded 37 genes including 13 protein-coding genes (PCGs), 22 transfer *RNAs*, two ribosomal *RNAs* genes, and one control region. The total length of the PCGs was 11,295 bp. The start codons of PCGs were ATG (in COX2, ATP8, ATP6, COX3, ND3, ND4, ND5, ND6, and Cytb), ATA (in ND1 and COX1), ATT (in ND2) and GTG (in ND4L). The stop codons of PCGs were TAA (in ND4L and Cytb), TAG (in ND2 and ATP8), AGA (in COX2, ND5, and ND6), AGG (in COX1), and the incomplete stop codon T- (in ND1, ATP6, COX3, ND3, and ND4). The longest spacer region was 246 bp and it located between ND5 and ND6, which was also found in *Rana* cf. *chensinensis* (Li et al. 2016a). The gene arrangement was identical to the gene order pattern of *Rana* (Huang et al. 2019; Jiang et al. 2020; Suk et al. 2021). The overall nucleotide composition of A, T, C, and G in majority-strand was 28%, 29%, 29%, and 14%, respectively.

To explore the phylogenetic relationship of *R. johnsi*, a total of 28 mitogenomes were analyzed, including 23 mt genomes of *Rana* downloaded from NCBI (Lin et al. 2014; Ni et al. 2016; Li et al. 2016a, 2016b; Liu et al. 2017; Chen 2018; Yang et al. 2018; Huang et al. 2019; Jiang et al. 2020; Wang et al. 2020; Suk et al. 2021; Xiong et al. 2021) and four mt genomes of *Odorrana schmackeri*, *O. livida*, *Amolops hongkongensis*, and *A. wuyiensis* (Zhang et al. 2018) as outgroups (Figure 1). To align the 13 PCGs, we used Clustal W in Mega version 7.0 (Kumar et al. 2016). The optimal partitions and best-fitting models (GTR + I + G) were generated by PartitionFinder version 1.1.1 (Lanfear et al. 2012) based on the Bayesian information criterion (BIC) (Schwarz 1978). The phylogenetic relationship was constructed based on 13 PCGs of the 28 species using Bayesian inference (BI) and maximum-likelihood (ML) methods *via* MrBayes version 3.1.2 (Huelsenbeck and Ronquist 2001) and RAxML version 8.2.0 (Stamatakis 2006), respectively. The BI and ML trees were showed the same topology (Figure 1). In BI and ML trees, subgenus *Lithobates* formed the basal clade within all other *Rana* species (Fouquette and Dubois 2014). Excluding the clade of subgenus *Lithobates*, *Rana draytonii* was the basal clade to other *Rana* species, which included the clade of *R. johnsi* as the basal clade. The species of *R. sangzhiensis* and *R. johnsi* were belonging to the genus *Pseudorana* (Dubois 1992), but both of them were located in different clades of *Rana* in this study. *R. sangzhiensis* had a sister relationship with the clade of *R. zhenhaiensis*, *R. dabieshanensis*, and *R. omeimontis*. In this study, the monophyly of *Pseudorana* was not supported. The removal of *Pseudorana* was also supported by the studies of Che et al. (2007) and Pyron and Wiens (2011). The phylogenetic relationships of the other *Rana* species were similar to the results of Chen (2018), Wang et al. (2020), and Suk et al. (2021). According to the importance of mt taxonomy and phylogenetic inference, our study provided a correct phylogenetic relationship of *R. johnsi*. The new mt genomes of *Rana* can give us a further understanding of phylogenetic relationships within *Rana*.

**Figure 1.**
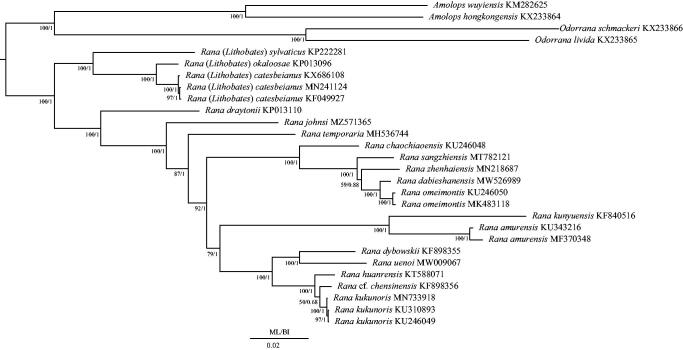
Phylogenetic tree of the relationships among 28 species of Ranidae including *Rana johnsi* based on the nucleotide dataset of the 13 mitochondrial protein-coding genes. Numbers around the nodes are the posterior probabilities of ML (left) and the bootstrap values of BI (right). The GenBank numbers and tribe of all species are shown in the figure.

## Data Availability

The genome sequence data that support the findings of this study are openly available in GenBank of NCBI (https://www.ncbi.nlm.nih.gov/nuccore/MZ571365) under the accession no. MZ571365.
